# The Zoonotic Risk of *Ancylostoma ceylanicum* Isolated from Stray Dogs and Cats in Guangzhou, South China

**DOI:** 10.1155/2014/208759

**Published:** 2014-05-04

**Authors:** Yuanjia Liu, Guochao Zheng, Muhamd Alsarakibi, Xinheng Zhang, Wei Hu, Liqin Lin, Liping Tan, Qin Luo, Pengyun Lu, Guoqing Li

**Affiliations:** ^1^College of Veterinary Medicine, South China Agricultural University, Guangzhou 510642, China; ^2^College of Animal Science, South China Agricultural University, Guangzhou 510642, China

## Abstract

Canine and feline hookworm infection is endemic in many countries with zoonotic transmission representing a potentially significant public health concern. However, there is limited data available on the zoonotic transmission of canine and feline hookworms in China. This study was conducted to evaluate the zoonotic risk of *Ancylostoma ceylanicum* isolated from stray dogs and cats in Guangzhou, south China. Primer pairs CAF/CAR were designed to amplify complete ITS sequences of obtained *A. ceylanicum*. The results were compared with fourteen ITS reference sequences of human-derived *A. ceylanicum* registered in GenBank, and phylogenetic trees were established by using NJ and ML methods. The sequence similarity of three dog-derived and five cat-derived *A. ceylanicum* with fourteen human-derived *A. ceylanicum* were 96.8%~100% and 97.8%~100%, respectively. Phylogenetic analysis placed *A. ceylanicum* isolated from dogs and cats in the same group with *A. ceylanicum* human isolates. Due to the ability of *A. ceylanicum* to cause a patent infection in humans, the zoonotic risk arising from dog and cat reservoirs to communities in this region should be determined.

## 1. Introduction

Hookworms are one of the most common parasitic nematodes which infect mammals including humans, dogs, cats, and wild canids [1]. They are prevalent in temperate and tropical regions of the world [2]. Currently, more than 100 species belonging to 18 genera of hookworms have been discovered, parasitizing the intestinal tract of carnivore and herbivore animal hosts.

*Ancylostoma ceylanicum* is the only species of canine and feline hookworms which can develop into adult form in the human intestinal tract, causing iron-deficiency anemia [3]. Parasitic to cats and dogs,* A. ceylanicum* is infective to humans as a zoonosis in some regions of Asia, but has not been associated with host blood loss in humans [4], and therefore was not considered a major pathogen [5]. In Malaysia, the prevalence of* A. ceylanicum* in humans was 23.4% with* A. ceylanicum* being the second most common species of hookworms [3], while one-third of human hookworm infection was caused by* A. ceylanicum* in Laos [6].* A. ceylanicum* was isolated from both humans and dogs in Thailand as well, and only in cases of human infection dyspepsia, abdominal pain, diarrhea, and other gastrointestinal symptoms was reported [7]. There were also reports of human infection with* A. ceylanicum* in Taiwan [8] and Fujian [9]. In Guangzhou, the infection rate of* A. ceylanicum* in stray dogs and cats was 42.67% [10] and 40.2% [11], respectively. Considering the zoonotic potential of* A. ceylanicum* infection between animals and humans, it is very important to evaluate the zoonotic risk of* A. ceylanicum*. The aims of this study were to amplify the complete ITS sequences of* A. ceylanicum* isolated from dogs and cats in Guangzhou, to compare the sequences of dog-derived and cat-derived* A. ceylanicum* with human-derived* A. ceylanicum*, and to construct a phylogenetic tree using ML and NJ methods to evaluate the zoonotic risk of* A. ceylanicum* between pet (dogs and cats) and humans.

## 2. Materials and Methods

### 2.1. Egg Samples

Egg samples of* A. ceylanicum* were collected through an epidemiologic survey of hookworm infections in dogs (*n* = 254, Guangzhou) and cats (*n* = 102, Guangzhou) in two previous studies [10, 11], preserved in 2.5% potassium dichromate and stored at 4°C.

### 2.2. Genomic DNA Extraction

Glass beads and proteinase K were added to 1.5 mL tube containing egg samples of* A. ceylanicum*. The suspension was thoroughly mixed with a vortex mixer for 30 minutes and then incubated at 55°C for 16 hours [11]. DNA purification was carried out according to the manufacturer's protocols.

### 2.3. PCR Amplification of the Complete ITS Sequence

The complete ITS was amplified using a forward primer CAF (5′-GACTGCGGACT GCTGTAT-3′) and a reverse primer CAR (5′-AAGTTCAGCGGGTAGTCA-3′) [11] that were derived from* A. ceylanicum* (GenBank: JQ812694, AJ920347, AM039739) and* A. ceylanicum* (GenBank: DQ381541, DQ780009). The PCR amplification was performed in 25 *μ*L volume containing 17.3 *μ*L of distilled water, 2.5 *μ*L of 10 × PCR Buffer, 2 *μ*L of diethylnitrophenyl thiophosphate (dNTP, 2.5 mM each), 0.5 *μ*L of each primer (CAF/CAR, 50 pmol/uL), 0.2 *μ*L of* Ex-Taq* polymerase (5 U/*μ*L), and 2 *μ*L of the DNA sample. The PCR reactions applied following cycling profile: initial denaturation of 96°C for 5 minutes, followed by 35 cycles of denaturation, annealing and extension at 96°C for 30 seconds, 60°C for 30 seconds, and 72°C for 60 seconds, respectively, and the final extension was carried out at 72°C for 7 minutes. The PCR products were analyzed with 1.0% agarose gel electrophoresis, at voltage 120 V for about 20 minutes. The results on the gel were observed under the UV transilluminator and recorded.

### 2.4. Purification and Sequencing of PCR Products

The positive PCR products by agarose gel electrophoresis were purified using UNIQ-10 column Cyclo-Prep PCR Clean-Up Kit. The purified products were sequenced by Huada Gene Biotechnology Co., Ltd. (Guangzhou).

### 2.5. Similarity Analysis of ITS Sequences

Homology analysis of* A. ceylanicum* ITS sequences from dogs and cats with fourteen ITS sequences of human-derived* A. ceylanicum* (GenBank: JF960362, JF960363, JF960365, JF960367, JF960368, JF960369, JN120871, JN120872, JN120874, JN120875, JN120876, JN120877, JN120880, and JN120881) was done using the MegAlign program of DNAStar (version 7, Madison, WI, USA).

### 2.6. Phylogenetic Analysis Based on ITS Sequences

The ITS sequences were aligned using the computer program CLUSTAL X 1.81. Phylogenetic trees were constructed by using neighbor-joining (NJ) and maximum likelihood (ML) methods employing MEGA 5.1 software (Arizona State University, USA). Two methods were carried out under default setting, and the consensus tree was obtained after bootstrap analysis, with 1000 replications.

## 3. Results and Discussion

### 3.1. PCR Amplification and Sequencing of Complete ITS Sequence

**Figure 1:**
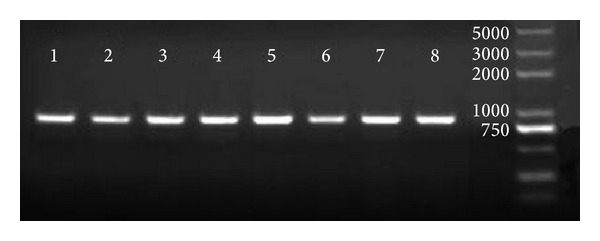
PCR amplification of ITS sequences of* Ancylostoma ceylanicum* from dogs and cats. Numbers 1–8 represent eight isolates: G23, G32, G21, M6, M55, M58, M60, and M76, respectively.

PCR amplification of the ITS yielded a fragment of 900 bp (Figure 1), and subsequent sequencing confirmed that the fragment contained partial 18S rDNA sequence, complete ITS sequence, and partial 28S rDNA sequence. The complete ITS sequence of three dog-derived* A. ceylanicum* isolates (G23, G32, and G21) and five cat-derived* A. ceylanicum* isolates (M6, M55, M58, M60, and M76) was 738 bp in length, containing ITS1 (364 bp), 5.8S rDNA (153 bp), and ITS2 (221 bp). The eight sequences had been submitted to GenBank (KC755027, KF279132—KF279138) respectively.

### 3.2. Similarity of ITS Sequences

**Figure 2:**
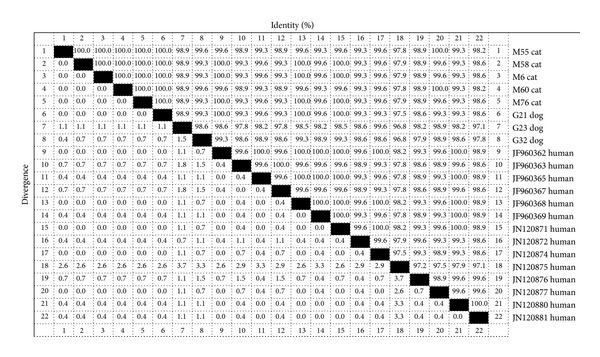
Homology alignment of dog- and cat-derived* A. ceylanicum* ITS sequence with human-derived* A. ceylanicum*.

BLAST results of dog-derived* A. ceylanicum* isolates (G23, G32, and G21) showed 96.8%~100% homology with 14 human-derived* A. ceylanicum*, where the homology of two dog isolates (G23, G32) with one human-derived* A. ceylanicum* isolate (GenBank: JN120875) was 96.8%, while one dog isolate (G21) was 100% homologous to three human-derived* A. ceylanicum* (GenBank: JF960362, JF960368, and JN120871) (Figure 2).

BLAST results from ITS sequences of five cat-derived* A. ceylanicum* isolates (M6, M55, M58, M60, and M76) were 97.8%~100% homologous with 14 human-derived* A. ceylanicum* isolates. Only the isolate (M55) was 97% homologous with the human-derived* A. ceylanicum* isolate (GenBank: JN120875), while several cat-derived* A. ceylanicum* isolates were 100% homologous with human-derived* A. ceylanicum* isolates (e.g., M55 with JN120877; M58 with JN120871, JF960368, JF960362; M6 with JF960368, JN120871, JF960362; M60 with JN120877; and M76 with JF960362, JF960368, and JN120871) (Figure 2).

### 3.3. Phylogenetic Analysis of ITS Sequence

Phylogenetic analysis of obtained ITS sequences, whether by NJ method or ML method, revealed a generally consistent topological structure of a phylogenetic tree, it showed that the clades of the six hookworms (*A. ceylanicum*,* A. braziliense*,* A. caninum*,* A. tubaeforme*,* A. duodenale*, and* Uncinaria stenocephala*) were obviously different. Based on the results of NJ method,* A. ceylanicum *had the closest genetic relationship with* A. braziliense*, but had a further genetic relationship with* A. tubaeforme*,* A. duodenale*, and* U. stenocephala*, while the ML method showed that* A. ceylanicum* had the closest genetic relationship with* A. caninum*,* A. tubaeforme*, and* A. duodenale*, but had the further genetic relationship with* A. braziliense* and* U. stenocephala*.

**Figure 3:**
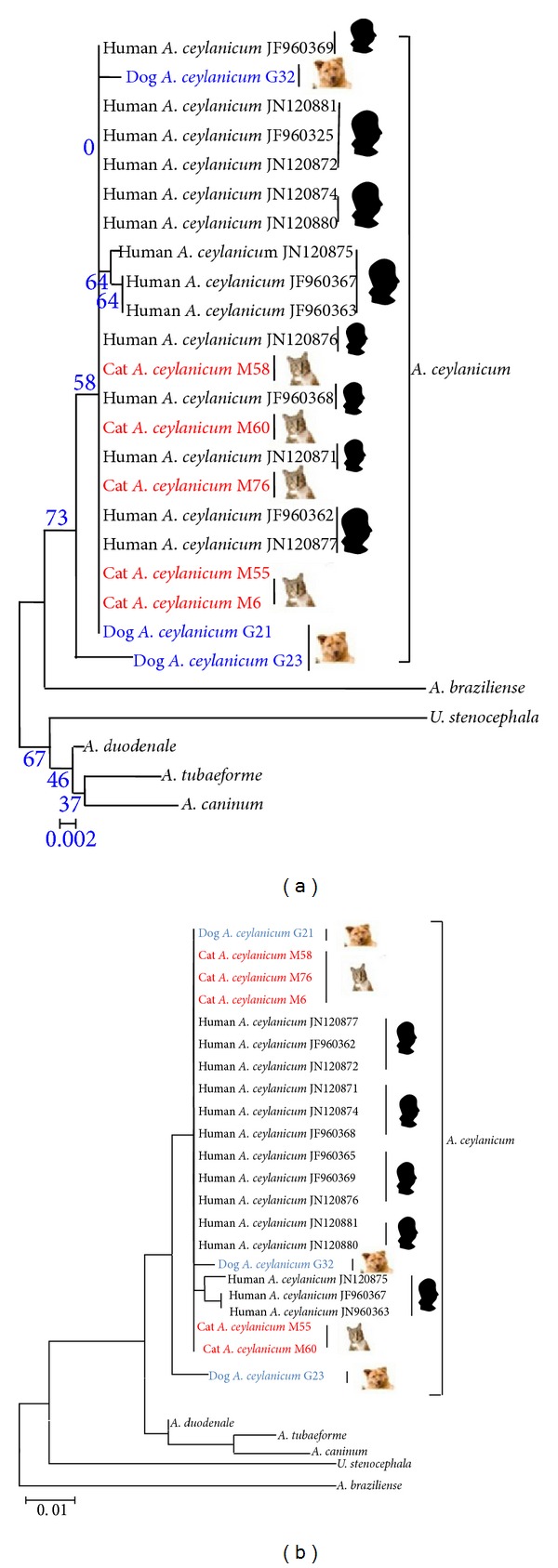
Phylogenetic relationship of hookworms from pets (dogs and cats) and humans based on ITS sequence by NJ (a) and ML (b) methods.

The phylogenetic tree constructed by NJ method revealed two major clades, where the dog-derived* A. ceylanicum* (G23) was located in a separate clade, but the other two isolates were clustered in a bigger clade, except the three human-derived* A. ceylanicum* isolates (JN120875, JF960367, and JF960363) which were grouped in a separate small clade, while other* A. ceylanicum* isolates were shown alternately in one clade, in the alternate state of the host. Similar structure were obtained by ML phylogenetic tree, where the isolate (G23) was located in a separate clade, and other* A. ceylanicum* isolates were located in the major clade as well, while the three human-derived* A. ceylanicum* isolates (JN120875, JF960367, and JF960363) were clustered in a small single clade. The remaining isolates of* A. ceylanicum* were located in one clade, which revealed an alternate state (Figure 3).

*Ancylostoma ceylanicum* was widely distributed in Asia (India, Taiwan, Thailand, Malaysia, Kalimantan, and Indonesia), Australia, South America (Suriname) [12, 13], and Africa [14]. Our results showed that the overall prevalence of* A. ceylanicum* infections in stray dogs and cats in Guangzhou (southern China) was up to 42.67% [10] and 40.2% [11], respectively, while Zhuang and Jin [15] reported 95.24% of* A. ceylanicum* in Guizhou Province (southwest China). Comparing a few authors,* A. ceylanicum* was less prevalent in Guangzhou than Malaysia (52% in dogs) [3] and Thailand (91% in dogs) [7]. This difference in prevalence would be due to the geographical distribution and development of Guangzhou city. In Australia, although anthelmintics were widely used for 10–15 years, it is shown that the prevalence of* A. ceylanicum* in dogs is still up to 10.9% [16], while Smout et al. [1] reported 100% prevalence of* A. ceylanicum* in wild dog scats in some rainforest regions of Australia.

Ngui et al. [3] posed that the infection of* A. ceylanicum* in both humans and animals living in the same endemic area had a strong correlation, as well as providing epidemiological and molecular evidence that supported the transmission of* A. ceylanicum* among human and domestic animals (dogs and cats). It is possible, therefore, that* A. ceylanicum* is the most abundant hookworm in tropical areas and that in locations where domestic dogs, the reservoir host of* A. caninum*, are present; there may be a spill-over of infection from domestic dogs that influences the hookworm species present in the wild dog population [1].

Our finding of high homology of ITS sequence between dog-, cat-, and human-derived* A. ceylanicum*, as well as the results of phylogenetic analysis, revealed the affinity in genetic evolution of* A. ceylanicum* from three different hosts, implying the zoonotic risk of* A. ceylanicum* between animals (cats, dogs) and humans. Thus, due to the ability of* A. ceylanicum* to cause a patent infection in humans, the zoonotic risk arising from dog and cat reservoirs to communities in the tropical regions should be determined. Future studies should also be carried out on animals to determine the extent of* A. ceylanicum* infection in Guangzhou and to assess the risk of zoonotic transmission and disease.
